# A Novel Bayesian Optimization-Based Machine Learning Framework for COVID-19 Detection From Inpatient Facility Data

**DOI:** 10.1109/ACCESS.2021.3050852

**Published:** 2021-01-11

**Authors:** Md. Abdul Awal, Mehedi Masud, Md. Shahadat Hossain, Abdullah Al-Mamun Bulbul, S. M. Hasan Mahmud, Anupam Kumar Bairagi

**Affiliations:** 1 Electronics and Communication Engineering DisciplineKhulna University 247289 Khulna 9208 Bangladesh; 2 Department of Computer ScienceCollege of Computers and Information TechnologyTaif University 125895 Taif 21944 Saudi Arabia; 3 Department of Quantitative SciencesInternational University of Business Agriculture and Technology 421876 Dhaka 1230 Bangladesh; 4 School of Computer Science and EngineeringUniversity of Electronic Science and Technology of China 12599 Chengdu 611731 China; 5 Computer Science and Engineering DisciplineKhulna University 247289 Khulna 9208 Bangladesh

**Keywords:** COVID-19, ADASYN, Bayesian optimization, classification, inpatient's facility data

## Abstract

The whole world faces a pandemic situation due to the deadly virus, namely COVID-19. It takes considerable time to get the virus well-matured to be traced, and during this time, it may be transmitted among other people. To get rid of this unexpected situation, quick identification of COVID-19 patients is required. We have designed and optimized a machine learning-based framework using inpatient’s facility data that will give a user-friendly, cost-effective, and time-efficient solution to this pandemic. The proposed framework uses Bayesian optimization to optimize the hyperparameters of the classifier and ADAptive SYNthetic (ADASYN) algorithm to balance the COVID and non-COVID classes of the dataset. Although the proposed technique has been applied to nine state-of-the-art classifiers to show the efficacy, it can be used to many classifiers and classification problems. It is evident from this study that eXtreme Gradient Boosting (XGB) provides the highest Kappa index of 97.00%. Compared to without ADASYN, our proposed approach yields an improvement in the kappa index of 96.94%. Besides, Bayesian optimization has been compared to grid search, random search to show efficiency. Furthermore, the most dominating features have been identified using SHapely Adaptive exPlanations (SHAP) analysis. A comparison has also been made among other related works. The proposed method is capable enough of tracing COVID patients spending less time than that of the conventional techniques. Finally, two potential applications, namely, clinically operable decision tree and decision support system, have been demonstrated to support clinical staff and build a recommender system.

## I. Introduction

The world is currently experiencing a pandemic situation due to the extensive spreading of the novel coronavirus disease namely, COVID-19. It is an acute respiratory syndrome triggered by the Severe Acute Respiratory Syndrome Coronavirus 2 (SARS-CoV-2), which was primarily detected in Wuhan under the Hubei province of China in late 2019. Considering the alarming rate of infection and death from the COVID-19, World Health Organization (WHO) announced the COVID-19 as a pandemic disease in March 2020 [1]–[3]. As per the WHO report on the COVID-19 on August 04, 2020, about 18,142,718 people have been infected due to COVID-19 [4]. Among them, about 691,013 people died so far. Due to its high contagious nature, both the COVID-19 infection and death toll are rapidly increasing.

In most cases, this disease spreads from man to man via respiratory droplets, transmitted from individual to individual via air or any other surfaces. This virus lives multiple hours to multiple days on a suitable surface at room temperature [5], [6]. As suggested by WHO, the COVID-patient should get himself isolated from others as early as possible to resist its transmission. The COVID-19 should be detected as early as possible, reducing life, livelihood, and the economy. But a critical issue is the broad maturation period of the COVID-19 that varies from 3 to 14 days. The usual symptoms of this disease include fever, cough, dyspnea, loss of smell, loss of taste, diarrhoea, etc. [7], [8]. People affected by COVID-19 should go through a fruitful, real-time, fast, and accurate screening scheme to ensure timely treatment, isolation, and safety for the patient.

Many pieces of research are going on to find out efficient and speedy COVID-19 detection schemes in different dimensions. The Reverse Transcription Polymerase Chain Reaction (RT-PCR) is a COVID-19 detection scheme that has shown its efficiency and has been practised worldwide. Using samples like the nasal or oral pharyngeal swab, this method can competently detect coronavirus and has attained the gold-standard banner. However, these testing kits fail to meet the mounting demand due to its limited supply, especially in developing countries [9]. Another drawback of this method is that it requires an extended period, ranging from one to two days. Moreover, the situation is even worse in rural areas, because people from remote areas get the results after two or more days, even after a week [10]. This extended period increases the vulnerability of the spreading of COVID-19 as the patient does not usually keep himself isolated from others before getting his result.

To optimize these limitations, the potentiality of Artificial Intelligence (AI) and Machine Learning (ML) algorithms in the analysis, characterization, and classification of different diseases have motivated the researchers to introduce AI and ML in COVID-19 detection. Numerous researches have already been carried out to design a COVID-19 detection model based on AI and ML [7]–[20]. Furthermore, Rajaraman and Antani [10] proposed a COVID-19 detection model based on deep learning (DL) algorithms. Using convolutional neural networks (CNNs), chest X-ray (CXR) data from patients are analyzed in this model to evaluate the presence of the SARS-CoV-2 virus. The model showed about 93% accuracy employing the VGG16 classifier. Another DL and CNNs based automatic COVID-19 detection model was proposed by Makris *et al.* [8]. Diagnosing the CXR data, the model exhibited about 95.9% and 95.00% accuracy engaging VGG16 and the VGG19 classifiers, respectively. A transfer learning-based model was presented by Abbas *et al.* [12] to trace COVID-19. This CNN based model diagnosed the CXR images of patients to check the COVID-19 presence, and the model attained about 97.5% accuracy. He *et al.* [7] presented a DL model for the automatic detection of COVID-19. This model employed the chest computed tomography (CT) images from patients to detect COVID-19. The anticipated 3D CNN model, MNas3DNet41, revealed about 87% accuracy. Jim *et al.* [11] presented an automatic COVID-19 detection model based on sequential CNN. This model took the CT images in its input to detect COVID-19. The model attained almost 92.5% accuracy along with 94.2% sensitivity and 95.6% specificity. A lot of more automatic COVID-19 detection models have been proposed so far based on the computer-based diagnosis of the CT and CXR images.

Hence, all the anticipated models require CT or CXR data of patients as the key input parameter, only available from diagnostic centres. So, each patient or suspected patient has to visit the diagnostic centre in person to check the presence of COVID-19 in his body. Most of the families in developing countries do not have private transport. Besides, patients from rural areas have to travel a long distance to reach a diagnostic centre. Therefore, they have to use public transport to visit the diagnostic centre to check COVID-19. This will create high vulnerability to COVID-19 spreading, among others. From another point of view, a low percentage of people tested for COVID-19 gets COVID-positive results in most of the countries; as an example, as of July 30, 2020, the positive rate is about 1.30% in France, 22.20% in Bangladesh, 9.90% in Iran, 0.90% in Italy, 7.90% in the USA, 11.10% in India, 2.10% in Russia, and 0.40% in the UK [21]. Visiting the diagnostic or test centre, a large percentage of COVID-19 negative people may meet with COVID-19 positive patients, which will enhance the risk of getting contaminated by COVID-19 disease. So, an inpatient data-based COVID detection will be the best option to avoid these types of risks. Besides, this type of detection will be very user friendly, cost-effective, and time-efficient.

Considering all the above issues, we have proposed a fast and user-friendly model to detect the COVID-19 based on machine learning. A large volume of data on COVID-19 is available in different laboratories and test centres. The dataset comprises other features like age, temperature, pulse rate, systolic and diastolic pressure, fever, cough, loss of smell, runny nose, diabetics, loss of taste, asthma, etc., which are analyzed to design the automatic COVID-19 detection model. The most promising advantage of this model is that it is capable of detecting the COVID-19 within a few minutes as well as help the doctors take adequate precautionary measures while treating the COVID patients. Different classification algorithms such as Linear Discriminant Analysis (LDA), Quadratic-DA (QDA), Naive Bayes (NB), k-Nearest Neighbors (KNN), Decision Tree (DT), Random Forest (RF), eXtreme Gradient Boosting (XGB), Gradient Boosting (GB), Support Vector Machine (SVM), etc. are used to characterize the model. These classifiers have some hyper-parameters, and proper tuning of these hyper-parameter improves the performance of the classification using state-of-the-art global optimizers such as Bayesian optimization [22], Gradient-Based Optimizer (GBO) [23], Slime mould algorithm (SMA) [24], and Harris hawks optimization (HHO) [25] etc. The evaluation of different performance metrics such as accuracy, specificity, sensitivity, etc. for the anticipated model demonstrates higher efficiency in detecting COVID-19. The contribution and key topics covered by this study are as follows:

• The proposed model can be easily tested on inpatients or inhouse facilities discussed in Section II. Therefore, the patient needs not to visit the clinic to test the COVID-19.
• We have designed a machine learning framework using Bayesian optimization adapted by the ADASYN algorithm to detect COVID-19 which is presented in Section II.D and II.E.
• The state-of-the-art machine learning technique is optimized using our method and compared with other commonly used Grid-search and random search techniques; see Section III.H.
• The proposed method uses the ADASYN algorithm to balance the model, and the effect of ADASYN has also been demonstrated in III.A.
• Using SHapely Adaptive exPlanations (SHAP) analysis, important features are calculated, and the SHAP values are explained to interpret the model in Section III.F.
• A clinically operable decision tree is built that will be helpful for the clinical staff stated in Section IV.A. A decision support system has also been developed to assist the recommender system illustrated in Section IV.B.

The remainder of the paper is organized as follows. In Section II, we discuss the materials and methods used in this work. We present the experimental results in Section III. In Section IV, we present a systematic discussion and comparison of the work with other approaches. Finally, we draw some conclusions in Section V.

## II. Materials and Methods

### A. Data Source

**FIGURE 1.**
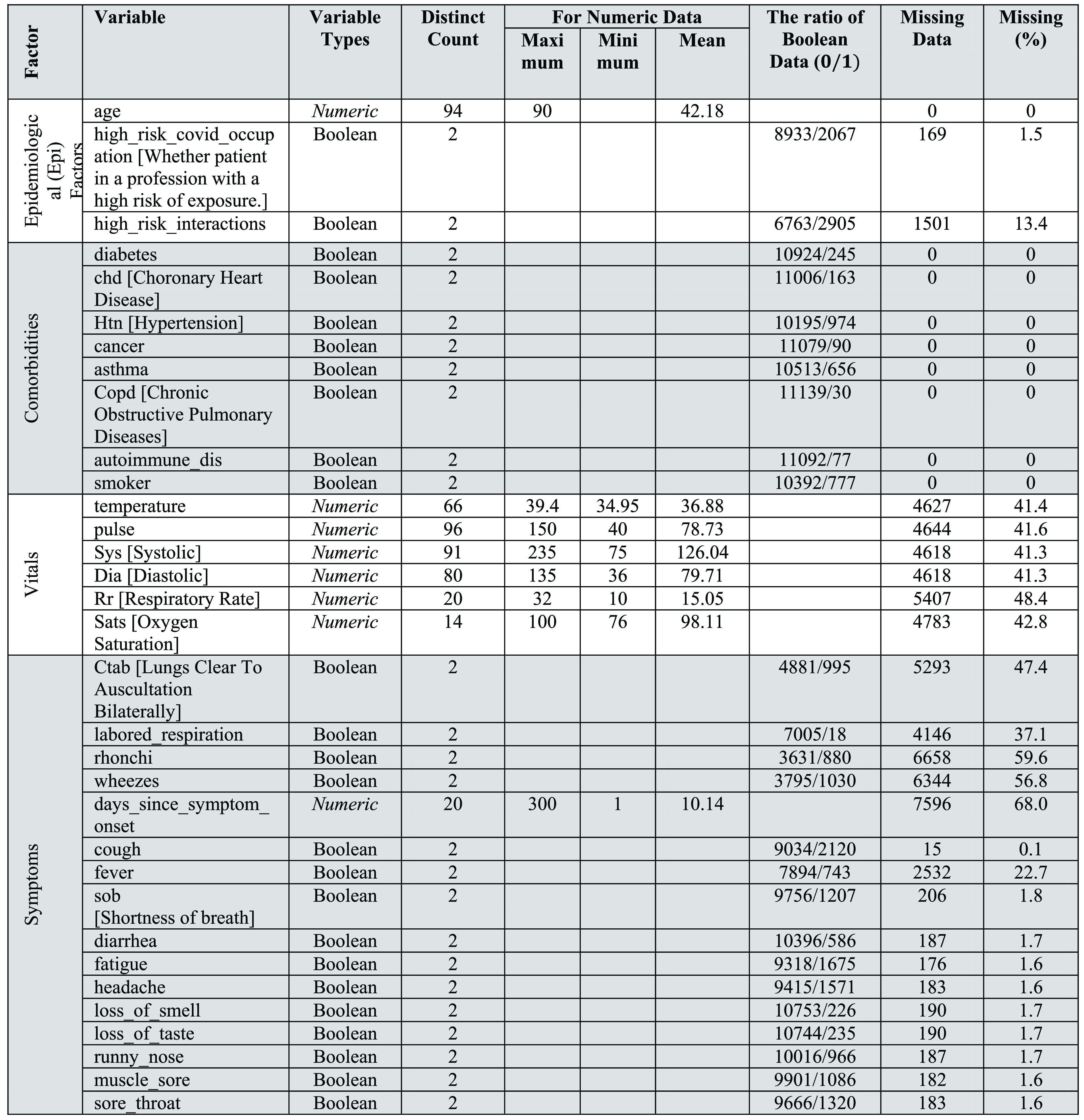
Characteristics of the Sample.

The clinically-driven information on individuals who have undergone through RT-PCR test was collected from the [26]. The dataset, containing 11169 person’s data with 2.82% of patients’ COVID positive and 97.18% COVID negative tests from the United States, was prepared by Carbon Health (CH) and Braid Health (BH). The CH started RT-PCR testing of a coronavirus in early April 2020. The dataset is compliant with the Health Insurance Portability and Accountability Act (HIPAA) privacy rule’s de-identification standard. Five clinical teams worked under the CH. The dataset prepared by the CH covered multiple physiognomies on patients, including Epidemiological (Epi) Factors, comorbidity, vital signs, lab technician-assessed symptoms, patient-stated symptoms. Whereas, two clinical teams gathered the dataset under the BH, which assembled the radiological information containing verdicts, CXR impressions, CXR labels, and CXR link. We haven’t used radiological information as most of the patient doesn’t have radiological details. The integration of radiological information is beyond the scope of this study, hence excluded from the analysis. The dataset consisted of both positive and negative test results for patients having both one or more symptoms and zero symptoms. In addition to COVID-19 test results, the complete dataset, available on the GitHub website, contains multiple features of patients such as pulse rate, temperature, age, higher danger introducer occupation, higher danger contacts, diabetics, cancer, asthma, smoker, systole, diastole, diarrhoea, fatigue, fever, losing smell, losing taste, runny nose, headache, muscle pain, pain in the throat, cough, shortness of breath, etc. The vignette of the entire data set has been illustrated through a tabular sketch shown in Figure 1.

**FIGURE 2.**
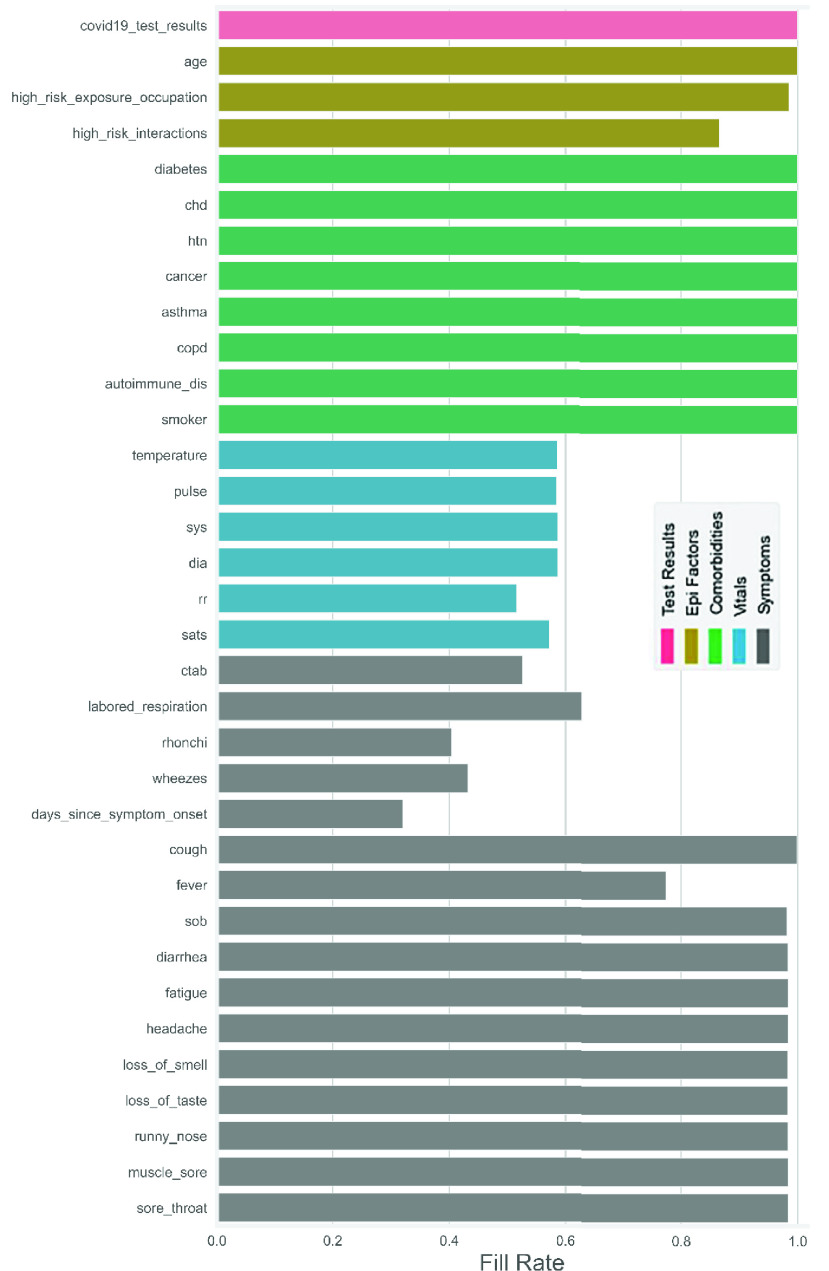
Fill rate for all Variables.

From the pictorial depiction (Figure 1), it is much clearer that there are two types of data (numeric and boolean), where the boolean variables are more than three times that of the numeric data. Moreover, the highest age of the patients in this data set is 90 years old, and the extreme values of both systolic and diastolic pressures were dramatically higher than the natural ones. It can be further added that $days\_{}since\_{}symptom\_{}onset$ has about 68% missing data, while the percentage of missing data in the entire data set is around 17. Besides the tabular display, as shown in Figure 1, the graphical example the green bars in Figure 2 efficiently reveals that the variables $cough$, $diabetes$, $chd$, $htn$, $cancer$, $asthma$, $COPD$, $autoimmune\_{}dis$, and $smoker$ have no missing data. In contrast, the variable $days\_{}since\_{}symptom\_{}onset$ has the highest missing values compared to others.

### B. Data Pre-Processing

**FIGURE 3.**
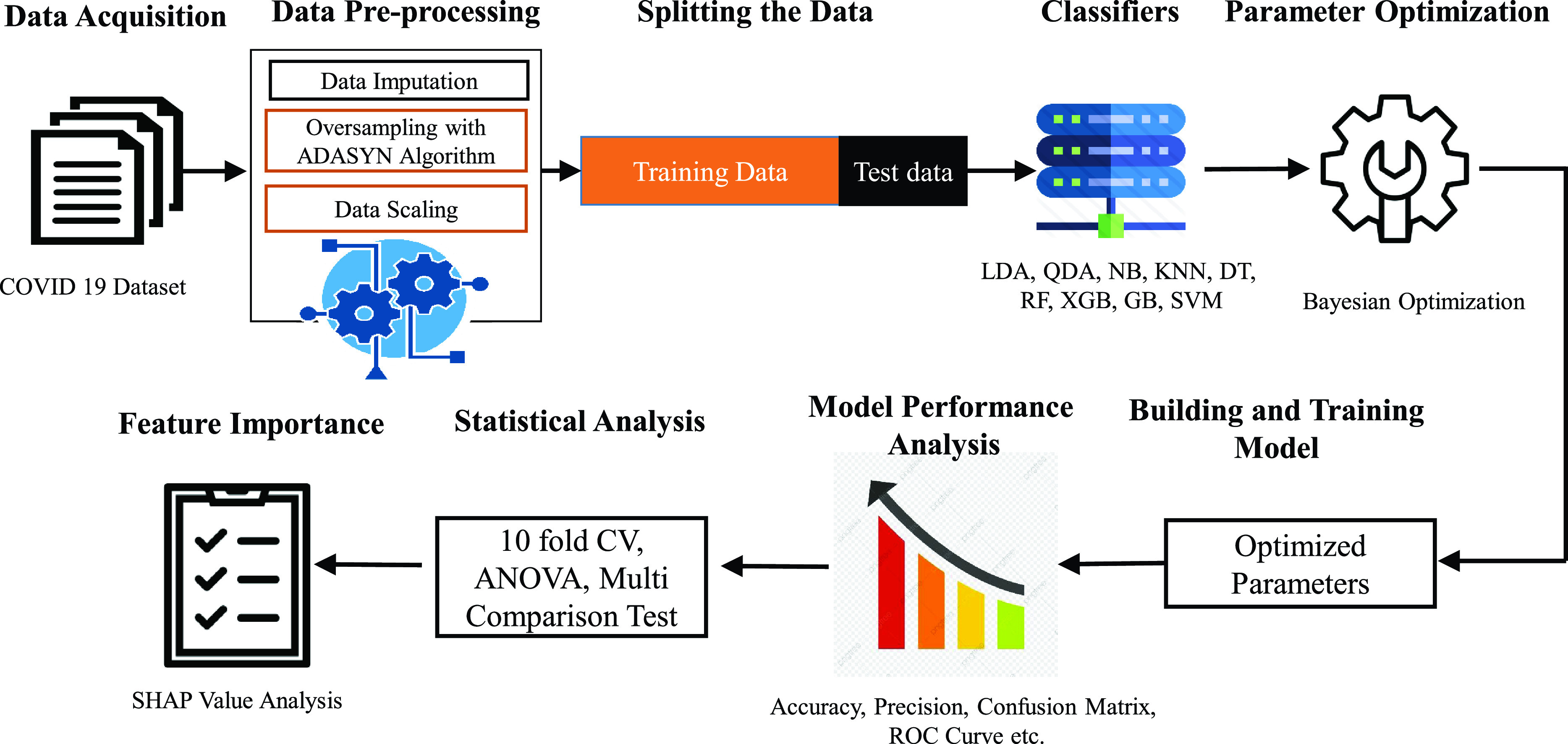
The overall workflow of the classification of COVID-19. The first phase is collecting raw data followed by pre-processing, where the raw data is imputed, scaled, and most importantly, the imbalanced data is balanced using ADASYN algorithm. Secondly, the pre-processed data are split into the train and test set used by different classifiers to measure the classification performance. In the next step, Bayesian optimization has been implemented to tune the hyperparameters of the classifiers. This optimized classification model is then tested, and different performance metrics (accuracy, precision, Confusion matrix, ROC, 10-fold cross-validation, ANOVA, and multi-comparison test) have been used for evaluation. Finally, the important features have been efficiently traced using SHAP analysis.

The overall workflow of our study is presented in Figure 3. For data pre-processing, the dataset has been imputed using Multivariate Imputation by Chained Equations (MICE) algorithm [27]. After completing scaling, we used the ADASYN algorithm to balance out COVID and non-COVID datasets. ADAptive SYNthetic (ADASYN) algorithm [28] is an oversampling method where COVID positive is a rare instance. It helped us generate synthetic data, solving the over-fitting problem. In contrast, the under-sampling process is not the right choice in COVID classification. The majority class (i.e. COVID-no class) is downsampled to the amount minority class (i.e., COVID-yes). This process will reduce the amount of data that drastically cause data inefficiency, and it loses the vital information of COVID-no class. Our COVID data set is not a big dataset, and downsampling could mislead the diagnosis and detection. Compared to other correlated over-sampling methods such as AdaBoost in conjunction with Over/Under-Sampling and Jittering of the data (JOUS-Boost), Synthetic Minority Over-sampling TEchnique (SMOTE), SMOTE-Boost and, DataBoost-IM (DataBoost IMbalanced) algorithm, ADASYN can balance the imbalanced dataset, for example, COVID-19 dataset by reducing the bias introduced by the imbalanced data distribution [28]. Besides, ADASYN shifts the decision boundary to harder to learn examples which ultimately improves the classification accuracy. These two objectives, i.e. (i) bias reduction and (ii) introducing harder to learn neighbourhoods examples, are accomplished through the dynamic weight adjustment and adaptive learning procedure [28].

The Mathematical explanation behind the ADASYN algorithm is given below:

For illustration, if $m_{l}$ and $m_{s}$ represent the majority and minority classes, respectively, then the Degree of imbalance of the two classes can be figured as follows:

\begin{equation*} d=\frac {m_{s}}{m_{l}}.\tag{1}\end{equation*}

If $d < d_{x}$ (where $d_{x}$ is the preset threshold for the maximum tolerated imbalance) then the total number of the synthetic minority can be estimated as follows:

\begin{equation*} G=(m_{l}-m_{s})d.\tag{2}\end{equation*}

Here $d = 1$ means there is a total balance between two classes. If $r_{i}=\frac {m_{l}}{k}$, where $k$ is the number of neighbours of each minority, and $\hat {r} = \frac {r_{i}}{\sum r_{i}}$ such that $\sum \hat {r}=1$, then the amount of synthetic data to generate for each neighbourhood can be calculated as:

\begin{equation*} G_{i}=G\hat {r}.\tag{3}\end{equation*}

If $x_{i}$ and $x_{u}$ are two minority examples within the same neighbourhood, where $x_{u}$ is randomly selected, then the new synthetic example, $s_{i}$ can be enumerated using the followings:

\begin{equation*} s_{i}=x_{i}+(x_{u}-x_{i})\lambda,\tag{4}\end{equation*}

where $x_{u}{-} x_{i}$ is the difference vector in $n$-dimensional spaces, and $\lambda $ is a random number over [0, 1].

### C. Classification Models

These nine classifiers such as Linear Discriminant Analysis (LDA), Quadratic Linear Discriminant Analysis (QLDA), Naive Bayes (NB), KNN, DT, RF, XGB, GB, and SVM, have been utilized in the proposed machine learning framework. Among nine classifiers LDA, QLDA, NB, KNN, DT and, SVM are common classifiers and also used in COVID-19 classification. RF, XGB and GBC are recent state-of-the-art classifiers. For example, XGB is recently applied to interpret the mortality prediction in COVID-19 patient and proposed a clinically operable simple tree-based tool which can be suitable to take the right decision from an expert point of view [56]. Considering the above rationale, we have used both commonly used classifiers as well as recently updated classifiers in this study. This will allow us to compare the classification performance in different classifiers. Moreover, RF, XGB and GBC classifiers can be explained through SHAP analysis which is very useful to clinical engineers. Finally, it can be seen from the results that the XGB performed better in most of the classification metrics, and we used SHAP to explain the XGB to interpret the COVID-19 detection.

#### 1) Linear Discriminant Analysis (LDA)

The LDA, introduced by Ronald Aylmer Fisher in 1936 [29], is a productive classification technique. It sorts-out n-dimensional spaces into 2-dimensional spaces that split-up by hyper-plane. The core objective of LDA is to trace the mean function for each class, and the function is projected on the directions that optimize between-groups variance and reduces within-group variance. The LDA is generated from the conditional distribution of the data $P(X|Y=k)$ for each class $k$, and it optimizes by taking the class k when the measurements are made on standalone variables for each observation are continuous quantities [30], [31].

#### 2) Quadratic Linear Discriminant Analysis (QLDA)

QLDA, an extension of LDA is exploited in machine learning and statistical analysis to classify two or more groups by quadratic discernible using distance-based classification techniques. There is no hypothesis like LDA that the covariance matrix for every class is identical. When the regularity hypothesis is true, the best prospective test for the hypothesis that an assumed measurement is from a given class is the likelihood ratio test. QLDA can be found from the conditional distribution like LDA of the data $P(X|Y=k)$ like LDA, and it maximizes by selecting the class $k$ [30], [31]. More precisely, for LDA and QLDA, $P(X|Y=k)$ is resulting as a multivariate Gaussian distribution with the following equation:

\begin{align*}&\hspace {-0.5pc}(Y=k) = \left({(2\pi)^{d/2}\bigg |\sum _{k}\bigg |^{1/2}}\right)^{-1} \\& \qquad\qquad\qquad \exp \left({-0.5(X-\mu _{k})^{t} \sum _{k}^{-1}(X-\mu _{k})}\right),\tag{5}\end{align*}

where $d$ is the number of features [32]. It needs to estimate the class priors $P(y=k)$ for using LDA and QDA model as classifiers, e.g. the proportion of instances of class $k$ from the training data, the means $\mu _{k}$ and the covariance matrix.

#### 3) Naive Bayes (NB)

NB classifier is authoritative and mainly useful in the large dataset. It is used in both machine learning and medical science, especially the diagnosis of different diseases like COVID-19. It is a Bayes’ theorem, based on probabilistic classifier objects with the strong independent supposition between the features. It generates conditional probability models that allocate class labels to a given problem [33]. Say,

\begin{align*}&\hspace {-0.5pc}P(\textrm {Patient}|\textrm {Covid Positive}) \\& \qquad\qquad\qquad {= \frac {P(\textrm {Covid Positive}|\textrm {Patient})\times P(\textrm {Patient})}{P(\textrm {Covid Positive})},} \end{align*}

where, $P(\textrm {Patient}|\textrm {Covid Positive})$, a conditional probability is the likelihood of the patient occurring that s/he is affected with Covid; $P(\textrm {Covid Positive}|\textrm {Patient})$ is also a conditional probability: the likelihood of the positive COVID occurring that is truly a patient; $P(\textrm {Patient})$ is the prior probability of a patient; $P(\textrm {Covid Positive})$ is the overall probability of observing COVID positive.

#### 4) K-Nearest Neighbours (KNN)

KNN is straightforward simplest algorithms in supervised machine learning technique [34] uses data and classify new data points based on similarity measures with the distance function, be able to apply to solve both classification and regression difficulty. It uses an integer number as 1, −1, or 0 for symbolizing the productivity (labels) of a classification algorithm outputs. KNN is a memory-based classifier; for example, it remembers all the training data-points to predict test data by computing the similarity between an input sample and each training instance. For a given new data point $x_{0}$, it finds the $k$ training points $x_{r}$,$r=1,\ldots,k$ closest in distance to $x_{0}$ and then classify using majority vote among the $k$ neighbors [32]. For selecting $k$, it conducts the KNN algorithm respective times with various values of $k$ and opts for the $k$ that reduces the number of errors accurately.

#### 5) Decision Tree (DT)

DT is a hierarchical flow chart like structure that generate some decision rules. The DT creates a model that predicts the target variable by learning the decision rule from the data feature [35]. The main hyper-parameters of DT are $criterion$, $max\_{}depth$, $max\_{}features$. In DT, “Gini” or “entropy” is used as a $criterion$. In contrast, the $max\_{}depth$ is utilized to limit the number of nodes in the tree, and the $max\_{}features$ represents the number of features to consider while searching for the optimal split. By properly tuning the hyper-parameters of DT (i.e., $criterion$, $max\_{}depth$, $max\_{}features$) applied on the COVID training dataset, the classification performance will be efficiently magnified.

#### 6) Random Forest (RF)

RF is an ensemble learning technique for classification that uses several DTs on different sub-samples of the dataset to improve the classification performance and to control over-fitting [36]. The main hyper-parameters of RF are $criterion$, $max\_{}depth$, $max\_{}features$, $n\_{}estimators$. The $criterion$, $max\_{}depth$, and $max\_{}features$ have already been discussed in DT. Besides, $n\_{}estimators$ represent the number of DTs in the forest. The performance of RF can be increased by properly tuning the hyper-parameters of RF through optimization.

#### 7) Gradient Boosting Classifier (GBC)

GBC is also an ensemble classifier that combines different weak learners (typically DT) into a single strong learner in a forward stage-wise fashion by optimizing the differentiable loss function [37]. Generally, ‘deviance’ or ‘exponential’ is used as a loss function where ‘deviance’ refers to deviance (logistic regression) for classification with probabilistic outputs. For thrashing, ‘exponential’ gradient boosting recaptures the AdaBoost algorithm. Other controlling parameters of GBC contained different parameters such as n estimators, learning rate, and max depth where n estimators indicate individual boosting stages to accomplish; learning rate reduces the performance of each tree [32].

#### 8) eXtreme Gradient Boosting (XGB)

XGB is designed based on the principles gradient boosting framework. It can be used for supervised learning tasks such as regression, classification, and ranking; similarly, it generates a prediction model in the form of an ensemble of weak prediction models. The model in a stage-wise approach is compassed with it as other boosting methods do, and it generalizes them by approving optimization of a random differentiable loss function. The gradient descent is used by ‘Gradient Boosting’ to generate new trees based on all previous trees. It supervises the objective function toward the minimum direction [38]. An objective function has a form, and it divides into training loss and regularization. The mathematical equation has been added as follows:

\begin{equation*} obj(\theta)=L(\theta) + \Omega (\theta),\tag{6}\end{equation*}

where $\theta $ denotes the parameters, $\Omega $ symbolizes the regularization term, and $L$ is the training loss. The main hyper-parameters of XGB are $N\_{}estimators$, $learning\_{}~rate$, $n\_{}jobs$, $max\_{}depth$, $Gamma$, $min\_{}child\_{}weight$, $colsample\_{}by\_{}tree$. The hyper-parameters such as $N\_{}estimators$, $learning\_{}rate$, $max\_{}depth$ have already been discussed. Besides, $n\_{}jobs$ are relevant to the number of parallel threads used to run XGB; $Gamma$ represents the loss required to make a further partition on a leaf of the tree. The $min\_{}child\_{}weight$ denotes the minimum sum of feature example, i.e., instance weight needed in a child, and $colsample\_{}by\_{}tree$ is used for the subsampling of columns.

#### 9) Support Vector Machine Classifier (SVC)

SVC is one of the most powerful supervised classifiers and used mostly for data classification in medical diagnosis [39], [40]. It aims to build a decision boundary in such a way that it is as far as possible from the closest data points from each of the classes, which are known as support vectors. For non-linear problems like COVID detection, a Radial Basis function (RBF) kernel is used. For RBF-SVC, the controlling hyper-parameters are Cost($C$) and Gamma($\gamma $). The Cost($C$) represents the regularization parameter that controls the misclassification of the training instances. Gamma($\gamma $) controls the “spread” of RBF kernel and, therefore, the decision region. The lower value of Gamma($\gamma $) will broaden the decision region and vice versa. The proper value of $C$ and $\gamma $ will increase the classification performance, which can be achieved by optimization.

### D. Requirement of Optimization

Most of the classifiers used in our entire study have some hyperparameters. The classifier itself is the function of hyperparameters, and these parameters control the hyper-plane. As an exemplification, XGB requires 7 Hyperparameters, while KNN and DT have one parameter each [Table 1]. Classifier performance indices, e.g., classification accuracy, error, specificity, sensitivity, etc. depend on the proper choice of these parameters. This is an optimization problem, whose general framework can be written as:

\begin{equation*} \lim _{z \in Z} J(Clf(z);Z),\tag{7}\end{equation*}

where $z \in Z$ denotes the hyper-parameters $z_{1},z_{2},z_{3},\ldots..{,}z_{n}$ belongs to $Z$. $Clf$ denotes the classifiers, e.g. RF, SVM, DT, NB, etc. and $J(.)$ represents the objective function. This objective function is the user-defined function where users can use different classifier metrics such as classification error or accuracy or other metrics described in the following section of statistical evaluation of classification measures. The general framework of the optimization problem can be interpreted as minimizing the classification objective $J(.)$ as a function of classifier’s hyperparameters $z \in Z$. In this study; mean of the the 10-fold cross-validation error is used as an objective function. We chose one of the state-of-the-art optimization algorithms named Bayesian optimization. This algorithm used a stochastic process, namely, as a Bayesian process, and it tried to find the optimal parameters in a smaller number of iterations saving both memory and time [41].

**TABLE 1:** Classifiers and Their Controlling Parameters or Hyperparameters

Classifiers (Clf)	Parameters, $z\in Z$	Hyper-parameters name
RF	4	Criterion, max_depth, max_features, n_estimators
RBF-SVM	2	Cost(C) and Gamma($\gamma $)
DT	3	Criterion, max_depth, max_features
NB	2	Alpha
GBC	5	Learning_rate, loss, max_depth, max_features, n_estimators
XGB	7	N_estimators, learning_rate, n_jobs, max_depth, Gamma, min_child_weight, colsample_by_tree
KNN	1	Number of Neighbours

Although various meta-heuristic algorithms such as GWO, GBO, SMA, and HHO etc. successfully integrated into many applications [42]–[44], hyper-parameter optimization in expensive-to-evaluate objective function e.g., 10-fold cross-validation loss, used in this study, makes it more complicated [45]. Besides, meta-heuristic algorithms require a set of input parameters that need to be found out to obtain an improved performance as the performance of the meta-heuristic algorithms are very sensitive to the input parameters. Furthermore, comparison among various meta-heuristic algorithms is only valid if the proper input parameters have been set, which requires domain knowledge [46]. Bayesian optimization is used to set the parameters of the meta-heuristic algorithm [45], [46].

The Bayesian optimization algorithm is a global optimization method that is specially designed to deal with such expensive-to-evaluate objective function, which is the population and genetic operator (mutation, cross-over, and selection) free algorithm. Bayesian optimization utilizes a Gaussian process to compute an acquisition function that evaluates the objective function. Besides, Bayesian optimization memorizes its previous evolution and utilize these statistics towards good solutions. It has been recently used in COVID-19 detection using x-ray images [22]. Considering the above rationale, Bayesian optimization has been applied in this study.

To justify further, the proposed Bayesian optimization is compared with the recently proposed Harris Hawk Optimisation algorithm [25]. This popular swarm-based and gradient-free optimization algorithm is based on the cooperative behaviour and chasing styles of Harris’ hawks in nature called “surprise pounce” [25]. We have chosen this algorithm for comparison as it is very recent and outperformed by many popular meta-heuristic algorithms such as GWO, Multi-Verse Optimizer, Moth-Flame Optimization, Whale Optimization Algorithm, Bat Algorithm, Cuckoo Search, Firefly Algorithm.

### E. Bayesian Optimization

Bayesian optimization (BO) is superior to grid search, random search, and manual tuning and therefore used in this study [47]. This algorithm keeps track of the past evaluation results and uses them to form a probabilistic Gaussian model of BO of the objective function and use it to find out the most optimal hyper-parameters; as an exemplar, in the case of RBF-SVM, the hyper-parameters are $C$ and $\gamma $. The BO algorithm selects $C$ and $\gamma $ for which objective function $J(RBF SVM;(C,\gamma))$ provides the minimum value. Note that, the classification error is used as an objective function. The BO algorithm is given below:

Step 1: Build a Gaussian probability model of the objective function. In this study, classification error is the objective function.
Step 2: Find the controlling parameters or hyper-parameters that perform best on the Gaussian process.
Step 3: Apply these hyper-parameters to the true objective function.
Step 4: Update the Gaussian model incorporating the new results.
Step 5: Repeat Step 2–4 until maximum iteration is reached.

The Mathematics behind the Bayesian Optimization for $X=(x_{1},x_{2},x_{3}, {\dots },x_{n})$ independent features and $y$ target variable is given below:

\begin{align*} P(y|X)=&\frac {P(X|y) P(y)}{P(X)}\tag{8a}\\=&\frac {P(x_{1}|y)P(x_{2}|y) {\dots } P(x_{n}|y) P(y)}{P(x_{1})P(x_{2}) {\dots } P(x_{n})}\tag{8b}\\=&\frac {P(y)\prod _{i=1}^{n}P(x_{i}|y)}{P(x_{1})P(x_{2}) {\dots } P(x_{n})}\tag{8c}\\=&\left[{\frac {1}{P(x_{1})P(x_{2}) {\dots } P(x_{n})}}\right]\times P(y)\prod _{i=1}^{n}P(x_{i}|y),\tag{8d}\end{align*}

Since all the variables except the target variable are independent, $P(x_{1})P(x_{2}) {\dots } P(x_{n})= Constant$, Then Eq. (8d) can be simplified as:

\begin{equation*} P(y|x_{1} x_{2} {\dots } x_{n})\propto P(y)\prod _{i=1}^{n}P(x_{i}|y),\tag{9}\end{equation*}

Now, from Eq. (9), we find the probability of a given set of inputs for all possible values of the target variable y and pick up the output with maximum probability:

\begin{equation*} y= \mathop {\mathrm {\arg \!\max }} _{y} P(y)\prod _{i=1}^{n}P(x_{i}|y),\tag{10}\end{equation*}

### F. Statistical Evaluation of Classification Metrics

We have used several performance evaluation metrics to evaluate the performance of the proposed framework. The accuracy (ACC), error, false-positive rate (FPR), sensitivity (SE), specificity (SP), positive predictive value (PPV), Matthew’s correlation coefficient (MCC), F1_score, and Kappa index can be calculated from confusion matrix [48], [49]. A lower value of error and FPR, and a higher value of ACC, SE, SP, PPV, MCC, F1_score, and Kappa index indicate a better model. Besides, 10-fold cross-validation has been used [52] on the overall dataset. The most significant point should be mentioned here that the box-plot and Analysis of Variance (ANOVA) test are typically executed, relying on the 10-fold cross-validation result. The statistical significance is determined by the p-value derived from the ANOVA test [50], [51]. Furthermore, the receiver operating characteristic (ROC) curve and the area under the ROC curve (AUC) has also been used to evaluate the performance of the classifier. The recall rate vs the decision boundary curve has been used to examine the performance. In this study, we have used the value of 0.5 as the decision boundary threshold to provide the same importance to COVID-yes and COVID-no classes.

### G. Feature Importance Using SHAP Values

The SHapely Adaptive exPlanations (shortly known as SHAP), proposed in recent papers by Lundberg and Lee [53], are calculated for any tree-based model. The SHAP values from Game Theory to attribute $\phi _{i}$ value to each feature can be mathematically ascertained using the following formula [54]:

\begin{equation*} \phi _{i}=\sum _{S\in N\setminus i}\frac {|S|!(M-|S|-1)!}{M!}[f_{x}(S\cup \{i\})-f_{x}(S)],\tag{11}\end{equation*}

where $M$ is the total input features, $N$ is the set of all input features, and $S$ is a subset of the input features.

• In this plot, all the variables are ranked in descending order.
• The horizontal line (x-axis) quantifies how much the value is associated with a higher or lower prediction. All the left-sided points represent the observations shifting the predicted value in a negative direction. In contrast, the points on the right contribute to shifting the prediction in a positive direction. All the features are on the left y-axis.
• The color shows whether that variable is high (in red) or low (in blue) for that observation.

## III. Experimental Results

In this paper, the Bayesian optimization has been used along with and without the ADASYN algorithm. In the case of ADASYN, sufficient adaptive synthetic data has been created to eliminate the imbalanced nature among the majority and the minority classes. Firstly, the effect of ADASYN has been evaluated along with ROC, shown in section III.A. The balanced model has also been tested on the original test data in section III.B. Box-plot and ANOVA are presented in section III.C using cross-validation accuracy to evaluate the statistical significance. The Recall rate vs. decision boundary curve and Bootstrap ROC with ADASYN are discussed in sections III.D and III.E, respectively. Then, the evaluation of feature importance using SHAP and the analysis of SHAP values have been presented in sections III.F and III.G, respectively. Finally, the performance of Bayesian optimization has been compared with the Grid search and random search in section III.H.

### A. Bayesian Optimization with and Without ADASYN

The newly obtained balanced dataset has been utilized; 67% of the total dataset is used for training and validation, and 33% is used for testing. After that, multiple classifiers are used, and various statistical measurements are presented. The effect of ADASYN has been experimented and validated in this subsection.

**FIGURE 4.**
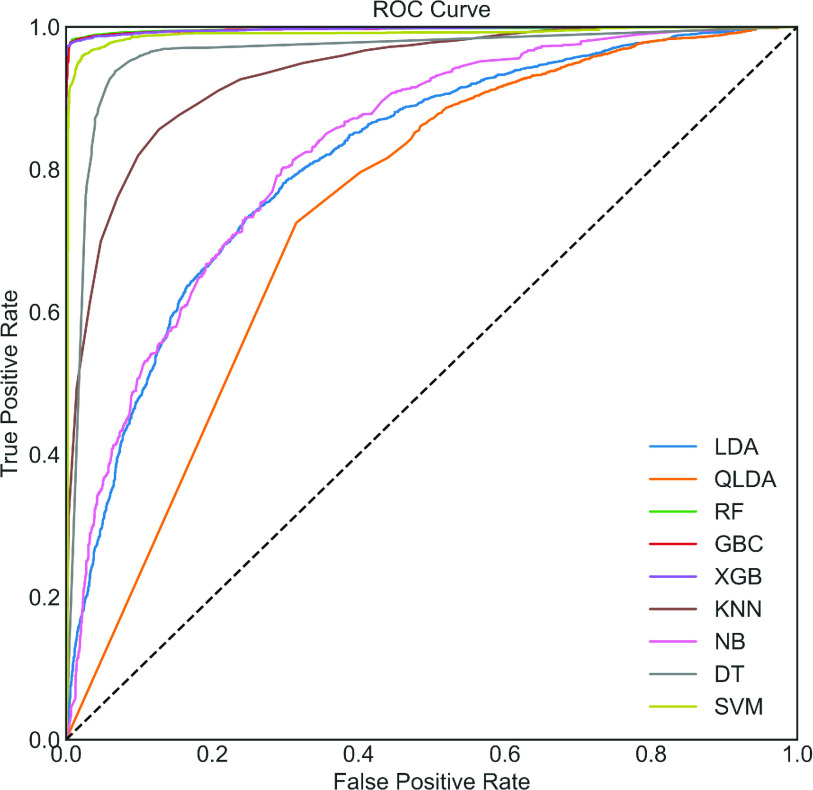
ROC Curve with ADASYN.

To begin, in the upper portion of Table 2, the performance analysis for the COVID Dataset with the utilization of the ADASYN algorithm has been demonstrated. It can be seen that; RF provides the highest classification performance. However, the performance of XGB and GBC is very close to RF. LDA and QLD show the worst classification performance among various classifiers presented in Table 2. The same AUC value of 99.70% is observed among these three classifiers, as shown in Figure 4. To demonstrate the effect of the ADASYN algorithm, the original unbalanced dataset is used. The dataset is also divided in the same manner, i.e., 67% of the total dataset is used for training and validation, and 33% is used for testing. We rerun the optimized code on this dataset, and the results on the test dataset without ADASYN is presented in the lower portion of Table 2. It can be observed that the highest accuracy of 97.17% is obtained by RF, which is close to the classification accuracy using RF with ADASYN. This could happen in the imbalance dataset. Therefore, accuracy is not a good performance indicator. The Kappa index, MCC, and AUC are more robust and reliable indicators in this case.

**TABLE 2:** Classification Performance (in %) on the COVID Dataset With and Without ADASYN

With ADASYN										
Classifiers	ACC	Error	F1_score	FPR	Kappa	MCC	PPV	SE	SP	AUC
$LDA$	73.69	26.31	75.11	31.55	47.35	47.60	71.68	78.88	68.45	81.30
QLDA	53.89	46.11	16.83	0.93	8.29	18.90	90.96	9.27	99.07	73.90
KNN	80.01	19.99	76.52	4.51	60.10	63.20	93.56	64.72	95.49	93.50
NB	73.74	26.26	75.60	33.48	47.43	47.91	70.98	80.87	66.52	82.70
DT	93.53	6.47	93.55	6.25	87.06	87.06	93.80	93.31	93.75	96.20
RF	98.59	1.41	98.61	2.08	97.19	97.20	97.97	99.26	97.92	99.70
XGB	98.50	1.50	98.52	2.30	97.00	97.02	97.76	99.29	97.70	99.70
GBC	98.50	1.50	98.52	2.02	97.00	97.01	98.02	99.02	97.98	99.70
SVC	96.60	3.40	96.63	3.51	93.20	93.20	96.54	96.71	96.49	98.90
Without ADASYN										
LDA	96.57	3.43	98.25	84.21	19.11	20.14	97.58	98.93	15.79	76.20
QLDA	17.96	82.04	27.22	7.37	0.56	3.86	98.66	15.79	92.63	68
KNN	97.14	2.86	98.55	100.00	0.06	0.30	97.16	99.97	0.00	64
NB	96.06	3.94	97.99	91.58	8.96	9.36	97.36	98.62	8.42	71
DT	97.14	2.86	98.55	100.00	0.06	0.30	97.16	99.97	0.00	57.60
RF	97.17	2.83	98.56	98.95	1.95	6.94	97.19	99.97	1.05	71.90
XGB	97.14	2.86	98.55	100.00	0.06	0.30	97.16	99.97	0.00	75.80
GBC	97.14	2.86	98.55	100.00	0.06	0.30	97.16	99.97	0.00	74.20
SVC	97.14	2.86	98.55	100.00	0.06	0.30	97.16	99.97	0.00	63.40

**FIGURE 5.**
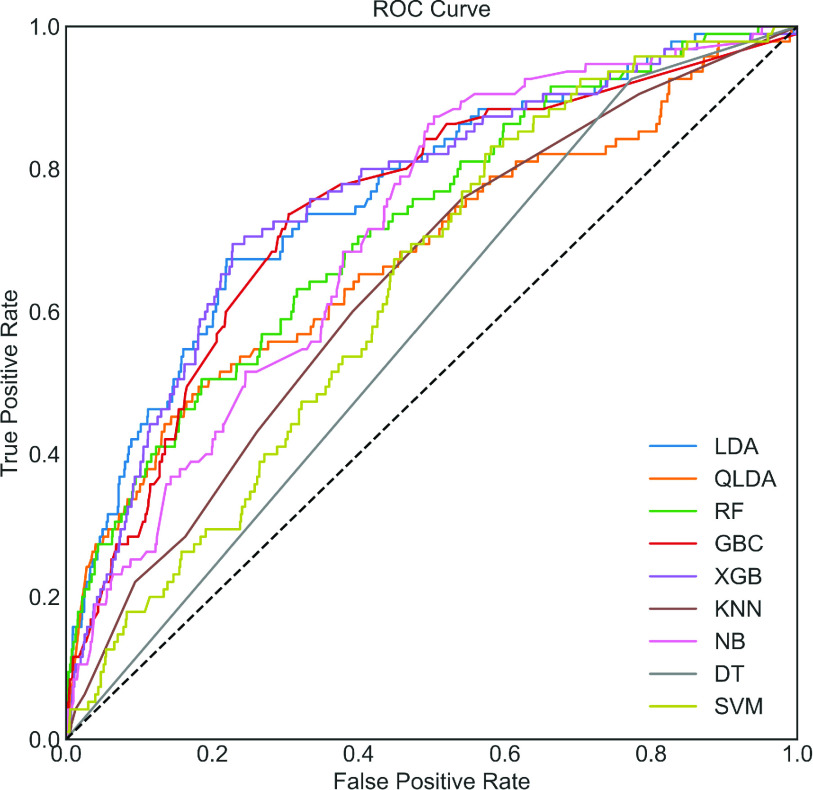
ROC curve without ADASYN. Note that the optimized model has not been created by using a balanced dataset.

It can be seen that the highest Kappa, MCC, and AUC values of 8.96%, 9.36% using NB, and 75.80% using XGB (Figure 5), respectively, are obtained. Compared to the upper portion of Table 2, i.e., results with ADASYN, the Kappa, MCC, and AUC values are 88.23%, 87.84%, and 23.90% times lower ADASYN algorithm is not applied, respectively. This can be happened due to an imbalanced model. This significant improvement using ADASYN concludes that classification performance can significantly be improved through directly applying the ADASYN algorithm.

### B. Results Using Original Test Data Only

So far, we have seen the effect of ADASYN on classification performance. The ADASYN is an oversampling method, and the synthetic data is mixed with original test data during data balancing. Therefore, it could be argued that what are the results of the balanced model on the original test data only where synthetic data is not mixed?

**FIGURE 6.**
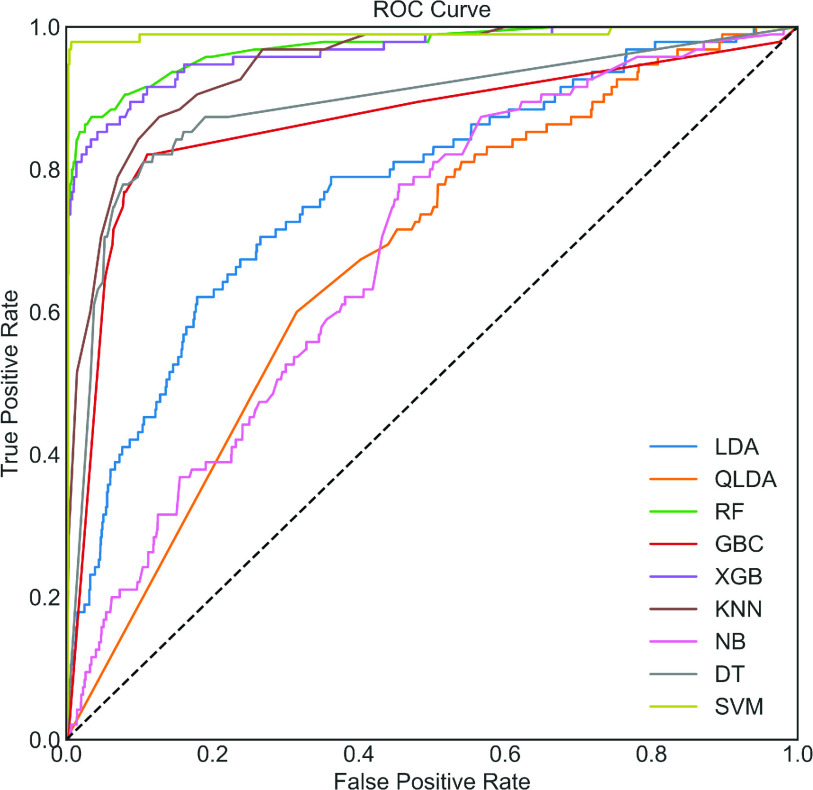
ROC Curve for COVID on original test data only using each model. The optimized model has been created by using a balanced dataset and then applied to the original test dataset.

To answer this question, balanced and Bayesian-optimized models have been applied to the original test data. Different performance measures, such as accuracy, sensitivity, specificity, and ROC, are presented in Table 3 and Figure 6. It can be seen that XGB provides the highest accuracy, error, F1_score, FPR, Kappa, MCC and sensitivity of 98.63%, 1.37%, 99.29%,24.21%,75.08%,75.08%, and 99.29%, respectively. In contrast, SVC provides the highest PPV, specificity, and AUC of 99.94%, 97.89% and, 98.90%, respectively. It can also be seen that XGB performs the best in most of the classification metrics presented in Table 3.

**TABLE 3:** Classification Performance (in %) on the Original Test Data of COVID

Classifier	ACC	Error	F1_score	FPR	Kappa	MCC	PPV	SE	SP	AUC
LDA	78.42	21.58	87.66	36.84	9.69	16.75	98.66	78.87	63.16	77.20
QLDA	11.82	88.18	16.97	1.05	0.51	4.76	99.67	9.28	98.95	67.20
KNN	65.62	34.38	78.53	3.16	8.98	21.16	99.86	64.71	96.84	94.40
NB	79.68	20.32	88.55	61.05	5.10	8.27	97.84	80.87	38.95	68.60
DT	93.38	6.62	96.50	27.37	35.65	40.97	99.16	93.98	72.63	88.80
RF	98.54	1.46	99.25	20.00	74.87	74.99	99.41	99.08	80.00	97.10
XGB	98.63	1.37	99.29	24.21	75.08	75.08	99.29	99.29	75.79	96.40
GBC	91.76	8.24	95.61	25.26	30.95	37.68	99.21	92.26	74.74	86.50
SVC	96.75	3.25	98.30	2.11	61.57	66.28	99.94	96.71	97.89	98.90

**FIGURE 7.**
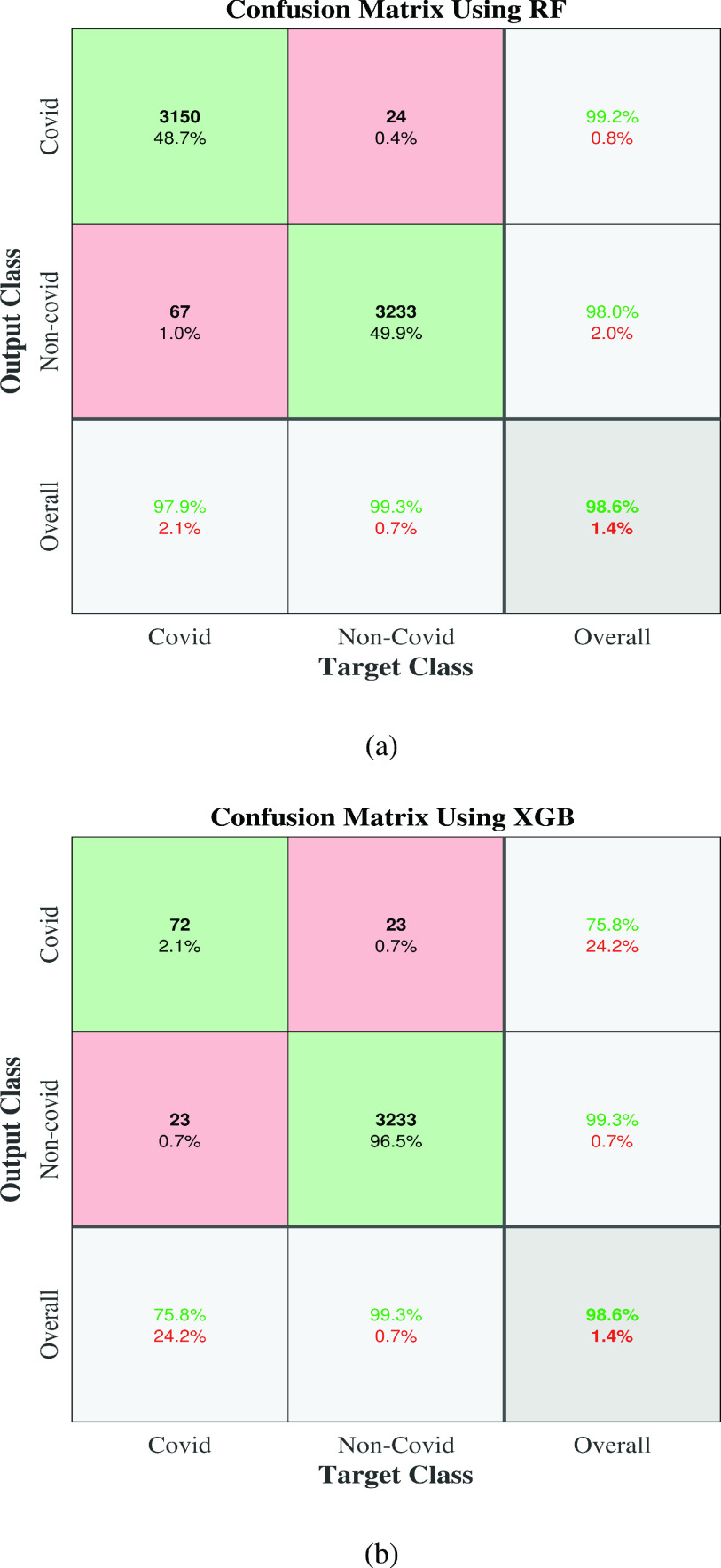
Confusion matrix of the balanced model applied in (a) COVID test Dataset with ADASYN, (b) original COVID test Dataset only. Figure 7(a) depicts the percentage of the correct classification in with the first two diagonal cells generated by the trained network. The numbers of patients who are correctly classified as a COVID and non-COVID were 3150 and 3233, corresponding to 48.7% and 49.9% in each group’s patients, respectively. Likewise, the numbers of patients who are incorrectly classified as a COVID and non-COVID were 24 and 67, with 0.4% and 1.0% correspondingly among all patients in each group. Similarly, the overall 99.2% were correctly, and 0.8% were incorrectly classified COVID, and non-COVID were overall, 98.0% and 2.0% correctly and incorrectly classified accordingly. In the case of prediction, the correct overall predictions for COVID and non-COVID were 97.9% and 99.3%, respectively. On the other hand, the incorrect results for COVID and non-COVID were 2.1% and 0.7%. Similarly, we can also interpret Figure 7(b).

Furthermore, these results are mostly inclined with ADASYN results (upper portion of Table 2), and results are significantly better than without ADASYN in all classification measures. The ROC curve shown in Figure 6 is also visually very close to Figure 4. Note that the same test dataset has been used without ADASYN (i.e., in the lower portion of Table 2) and in Table 3 for a fair comparison. Finally, it can be concluded that a balanced model can significantly improve the performance of the COVID dataset and XGB shows the best classifiers. The confusion matrix of the best performing balanced model with ADASYN and with original test data have been presented in Figure 7 to show how much COVID and Non-COVID patients are correctly classified.

### C. K-Fold Cross-Validation

In the standard train-test-split method, generally, a small portion of the data is taken as the test set, and the total dataset is not tested. To overcome this issue, the k-fold cross-validation (CV) is one of the helpful techniques exploited to test the effectiveness of machine learning models. It is also a re-sampling procedure to evaluate, and $k=10$ is used in this study. The first fold is used for testing, and the remaining folds are used for training and repeated ten times to test the total dataset fold-by-fold basis. The 10-fold cross-validation result is presented in Table 4, where the classification result of each fold is shown. The final row provides the average classification accuracy of the 10-fold results. From the Table 4, it is observed that the least score has been obtained using QLDA, whereas the XGB touched the mountain point, grabbing a score of 96.70% and RF has attained an average accuracy of 96.46%. On the other side, the classification performance using Decision Tree, SVC, and GBC was less than XGB and RF but above 90%. Note that, the data processed by ADASYN is used only to train the classifier, but the original test is used during testing and performance comparison.

**TABLE 4:** The Accuracy Score (in %) of the Different Optimized Classifiers Using 10-Fold Cross-Validation

	LDA	QLDA	KNN	NB	DT	RF	XGB	GBC	SVC
Fold-1	76.03	20.39	68.16	82.11	89.98	95.44	95.98	89.09	94.10
Fold-2	75.45	20.16	69.62	83.96	89.61	96.33	96.24	92.03	95.25
Fold-3	78.87	20.05	67.68	80.75	91.23	96.87	96.69	92.66	95.17
Fold-4	78.23	20.88	67.47	80.91	91.40	96.95	96.77	92.12	95.61
Fold-5	76.28	23.37	69.29	82.27	90.15	96.96	96.96	91.67	95.70
Fold-6	76.81	19.88	66.97	82.27	91.76	96.42	96.60	91.14	94.36
Fold-7	78.27	20.48	66.19	79.79	89.18	96.60	97.23	89.54	93.92
Fold-8	77.15	22.76	68.19	81.81	91.31	96.42	97.22	90.23	95.43
Fold-9	77.06	21.77	67.38	81.45	91.22	96.06	96.42	90.68	95.70
Fold-10	78.09	24.51	67.62	80.59	90.16	96.60	96.87	89.27	94.72
Average	77.22	21.43	67.86	81.59	90.60	96.46	96.70	90.84	95.00

**FIGURE 8.**
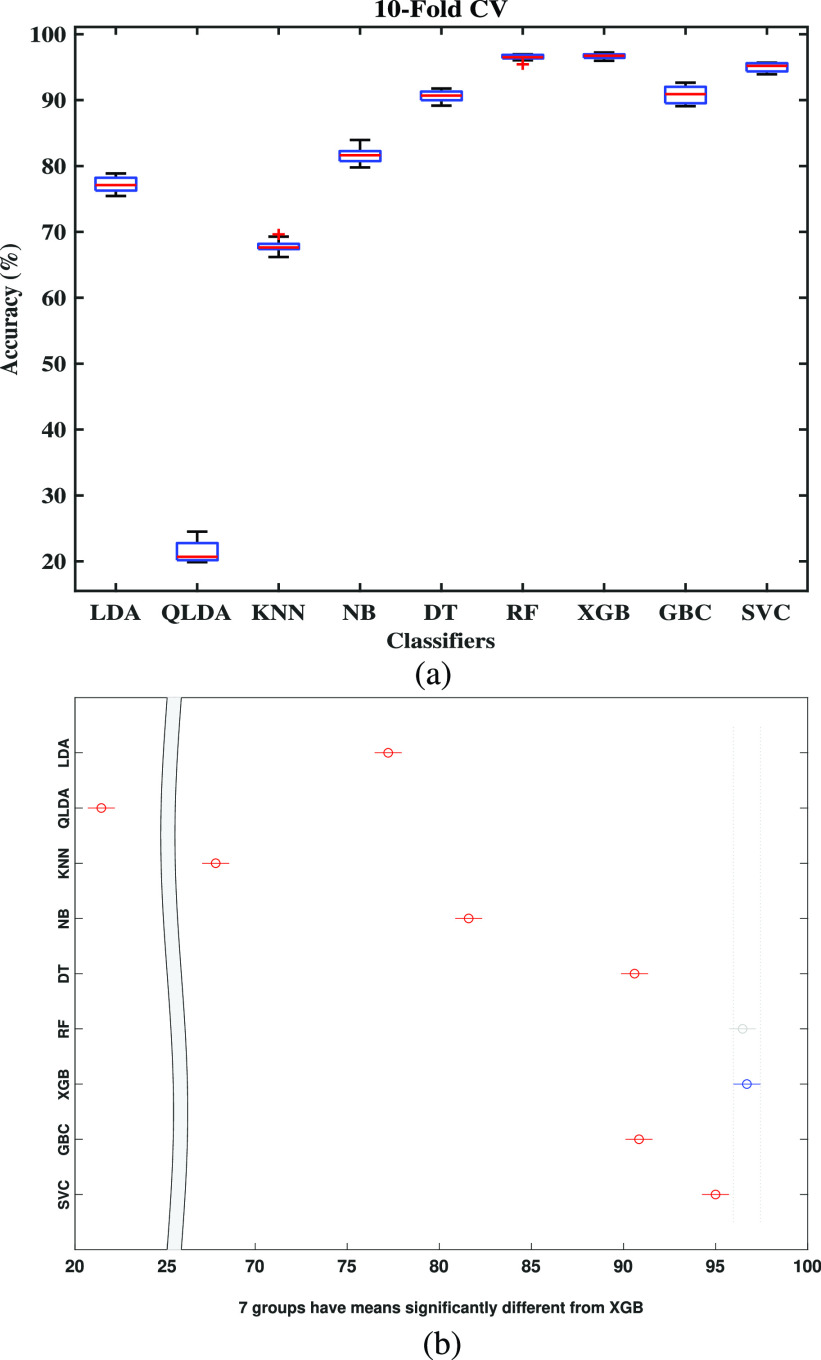
Box-plot for (a) COVID Dataset and (b) multi-comparison test. Note that (b) is a graphical user interface tool by which one can test the statistical significance of any classifiers. Here we only show the effect of XGB. The effect of other classifiers can also be interpreted in the same way.

Figure 8(a) showed the accuracy of different classifiers using the COVID original dataset using a box-plot. Here one-way ANOVA provided a $p$-value of $3.32 \times 10^{-107}$ for the original COVID test dataset, which is statistically significant ($p < 0.005$). It also provided an interactive plot of multiple comparisons of means in Figure 8(b) that showed the highest mean accuracy from XGB that is statistically significant from seven classifiers (GBC, DT, SVC, NB, KNN, QLDA, and LDA). In contrast, it is statistically not significant from RF, because the mean of RF is almost identical. Note that, Figure 8(b) is an interactive plot where the significance of different classifiers can be visualized by clicking on the specific classifier level. For instance, RF is blurred (shown in grey) defining its insignificance as XGB is selected. Similarly, GBC and DT will also exhibit statistical insignificance if one of them is selected.

### D. Recall Rate Vs. Decision Boundary Curve

**FIGURE 9.**
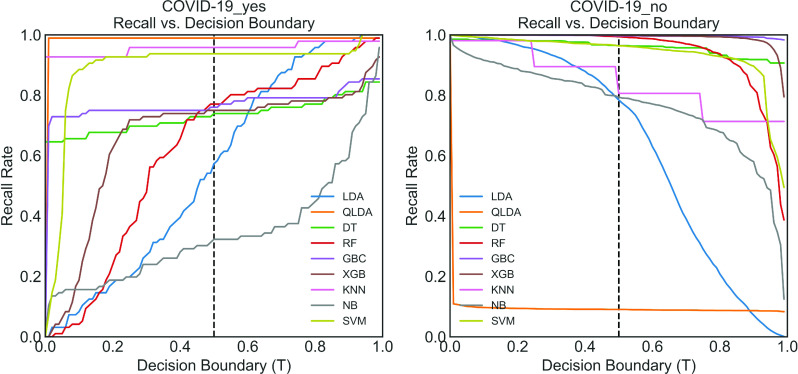
Recall rate vs. decision boundary curve for (a) COVID positive and (b) COVID negative.

The recall rate, in general, depends on the decision boundary using a certain threshold. To exemplify, the recall rate vs. decision boundary curve displayed in Figure 9(a), where 0.5 decision boundary threshold ($T$) has been used for the “COVID-19-yes” class. The recall rate of QLDA is about 0.98 at default threshold $T = 0.5$, meaning that about 98% times this optimized classifier can truly classify the “COVID-19-yes”. The XGB and RF provided a moderate performance of around 0.75 at default threshold T = 0.5 defining the “COVID-19-yes” class. The SVC shows the third highest performance of around 0.90. In contrast, the recall rate of NB at this threshold is 0.25, meaning that only 25% times NB can truly classify the “COVID-19-yes” class. A similar scenario is observed for the LDA classifier.

On the other hand, looking at Figure 9(b), the recall rate of QLDA is drastically falling to a value of 0.1 at $T = 0.5$, revealing that only 10% times QLDA can classify the “COVID19-no” class. The recall rate of XGB, GBC, and RF is about 0.99 at this threshold whereas the recall rate of SVC is 0.90. Finally, considering both “COVID19-yes” and “COVID-19-no” classification using recall rate vs. decision threshold measure, it can be concluded that SVC, XGB, and, RF provide the satisfactory recall rate among different optimized classifiers predicting both classes.

### E. Bootstrap ROC With ADASYN

**FIGURE 10.**
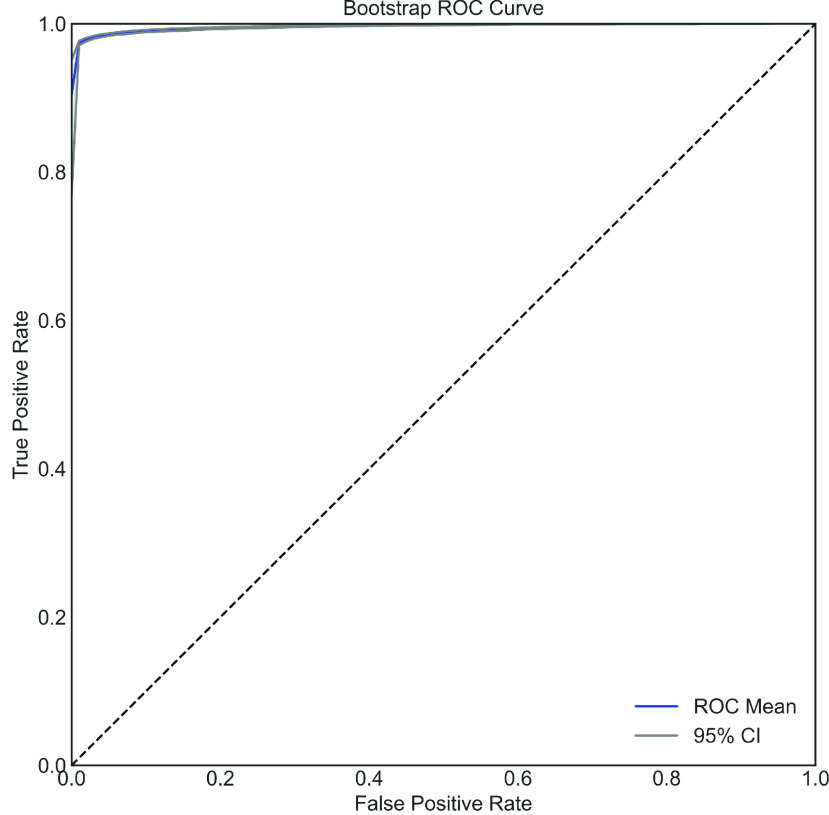
Bootstrap ROC curve of the COVID dataset using XGB with 95% CI.

To determine whether the optimized model is highly sensitive to training data or not, bootstrapping is performed on the XGB model as it is the best performing model. This gives $N_{boot}$ XGB having slightly different discriminative abilities. To show the error, three ROC curves are plotted in Figure 10; the middle one represents the average ROC where upper and lower curves represent the 95% confidence interval (CI). To obtain this bootstrap ROC, $N_{boot}=100$ XGB models are trained and mean AUC of 0.98 with an upper and lower confidence interval of 0.97 and 0.99, respectively, are obtained. This indicates that training is not highly sensitive to the training dataset.

### F. Feature Importance Using SHAP

**FIGURE 11.**
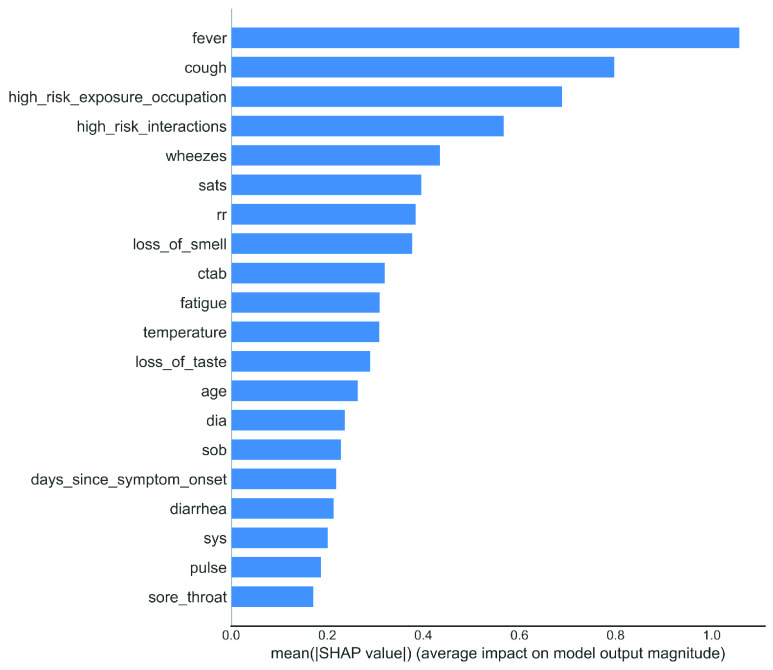
Feature importance plot using SHAP for XGB.

In a variable importance plot, the most significant variables are sorted in descending order. The top variables contribute more to the model than the bottom ones and thus have high predictive power. By way of example, “$fever$”, “$cough$”, “$high\_{}risk\_{}exposure\_{}occupation$”, “$high\_{}risk\_{}interactions$”, “$wheezes$” are the most important features, where “$fever$” touched the mountain point in this case [shown in Figure 11]. Simultaneously, “$pulse$” and “$sore\_{}throat$” received the least importance in classifying the COVID-19 contaminated patients.

### G. SHAP Value Analysis

**FIGURE 12.**
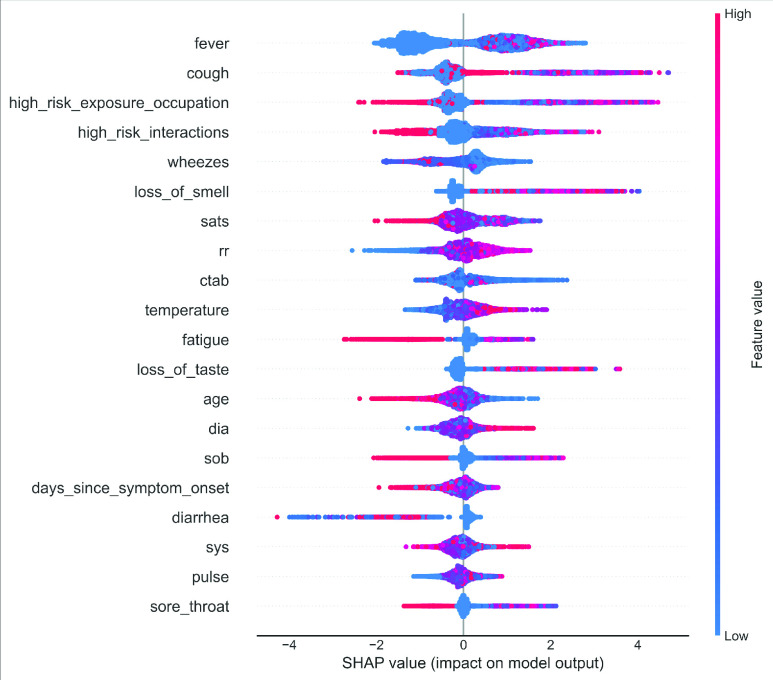
The SHAP variable importance plot of training data using XGB.

From the pictorial example of SHAP analysis [Figure 12] for training data, it can be summarized that the three features, “$fever$”, “$cough$”, “$high\_{}risk\_{}exposure\_{}occupation$” and “$loss\_{}of\_{}smell$” have a massive positive impact is on the target variable. The “high” comes from the red colour, and the “positive” impact is shown on the X-axis. Whereas, we conclude by mentioning that the features “$ctab$” and “$wheeze$” are highly negatively correlated with the target variable. In this way, all the variables can be efficiently explained. It should be mentioned that the behaviour of the XGB model is defined by the SHAP and are not necessarily causal in the real world. In other word, SHAP values do not provide the causality; it only describes the model behaviour and the behaviour of the data used to build the model [55]. As the model does not predict all the COVID patients accurately, it is plausible to get some false positives and false negatives. However, the SHAP value can able to explain such results, and the summary plot will be helpful to explain those results.

### H. Performance on the Grid Search, Random Search, Bayesian Optimization and Harris HAWKS Optimization

We propose to use Bayesian optimization techniques in our framework, and therefore, it is logical to compare the Bayesian optimization algorithm with commonly used parameter search algorithms. Two popular and widely used algorithms, namely, grid search and random search, compare with our proposed techniques. Table 5 presents the comparison of different search algorithms in terms of several parameters evaluated; the overall time is taken (in sec.) to complete the program, cross-validation accuracy score, test score. All the simulations were run on Intel core $i9$ computer having $64GB$ RAM and used the XGB model. It can be seen that it takes 10473.740 Sec. to complete the simulation using grid search, whereas random search and proposed Bayesian optimization take only 162.794 Sec. and 675.389 Sec, respectively. Furthermore, the random search and Bayesian algorithm take 30 parameters each, while the grid search requires more parameters, which is 218 times than that of others. The test score using Bayesian optimization is 98.20%, which is better than grid search, random search.

**TABLE 5:** Comparative Search Techniques

Optimization techniques	Parameters evaluated	The overall time is taken (in Sec)*	Cross-validation score (%)	Test score (%)
Grid Search	6561	10473.740	97.39	98.00
Random Search	30	162.794	97.97	98.10
Bayesian optimization	30	675.389	98.00	98.20
Harris Hawks optimization	200	6204.80	98.39	98.00
*In case of grid search: time is taken to iterate overall parameter combination.				
*In case of random search and Bayesian optimization: time is taken to go over a predefined number of iterations (30)				

**FIGURE 13.**
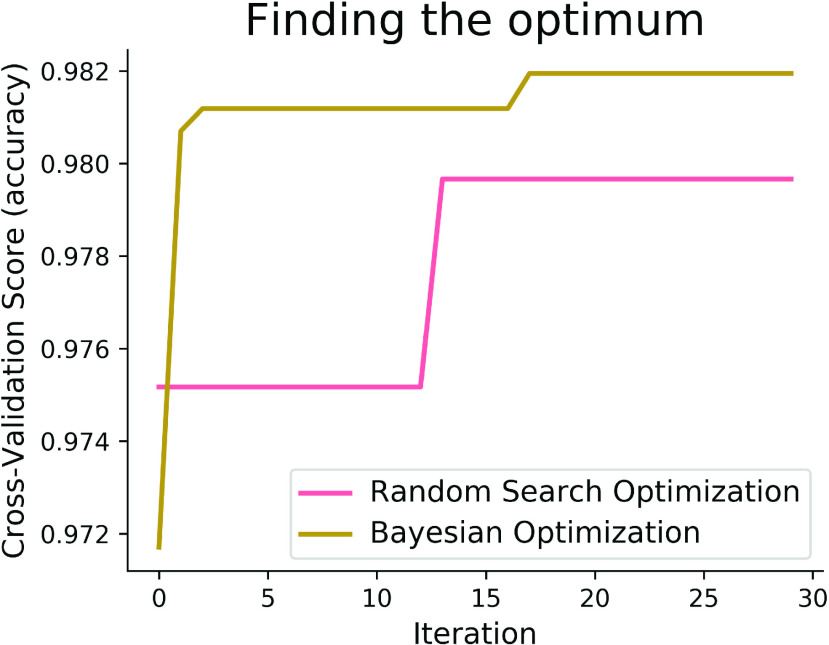
Comparative optimization techniques applied to the XGB model.

The pictorial depiction of the comparative search methods has also been given in Figure 13, from where it can be added that at the initial stage, the accuracy of Random Search was nearly 97.50%, which was almost stable up to 12 iterations. Then, with a single iteration, it takes a sharp change in its accuracy, touching closely the score of 98%, which was followed by an unchanged condition until 30 iterations. In contrast, the score of our proposed Bayesian Optimization technique commenced before 97%, which was almost steep up to 2 iterations, touching the accuracy above 98%. The most exciting information should be mentioned here that the score of our proposed method remains unchanged, except for a slight change after 15 iterations. Before finishing 30 iterations, its accuracy touched the mountain point.

The proposed Bayesian Optimisation Framework has also been applied to the most recent Harris Hawks optimization algorithm calculated over 200 evolutions with 20 populations on the same train-test settings. It provides 98.39% cross-validation accuracy, whereas the testing accuracy is 98%. The result is very similar to the Bayesian Optimisation framework. However, it takes 6204.80 Sec. which is 9.4 times slower than our proposed framework as it requires more evaluations and optimization calculations; see Table 5.

**FIGURE 14.**
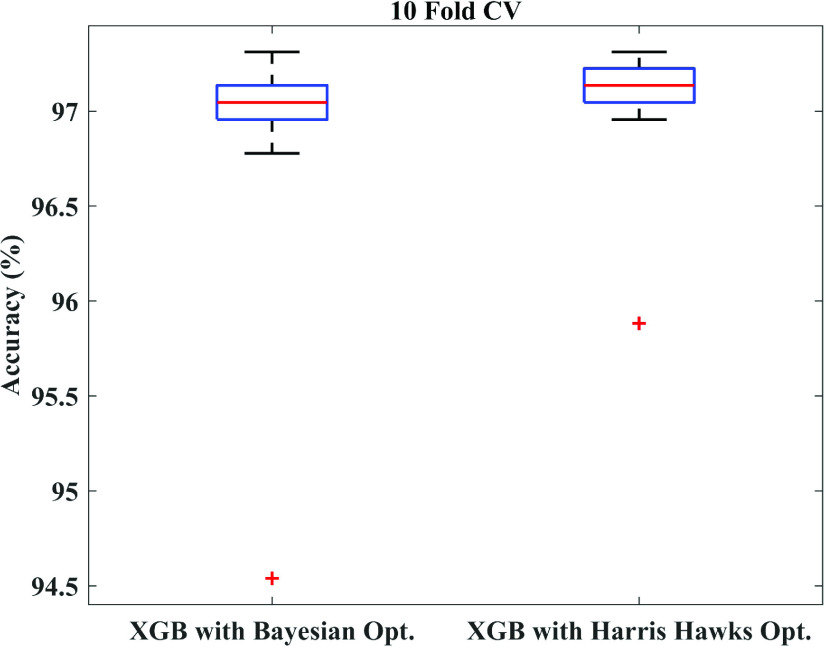
Box-plot of Bayesian optimization and Harris Hawks optimization.

To further justify, a statistical significance test between Bayesian optimization and Harris Hawks optimization algorithm is performed on 10-fold cross-validation using t-test. After that, the p-value is calculated, and the box-plot is plotted. A p-value of 0.47 is found, which suggests that there is no statistically significant difference between these two optimizations. The box-plot illustrated in Figure 14 also justifies the same statements.

## IV. Discussion and Comparison

In this research, a Bayesian optimization-based machine learning framework with a class balancing strategy using the ADASYN algorithm is proposed to identify COVID patients from their inpatient facility data. Nine state-of-the-art classifiers such as LDA, QLDA, NB, KNN, DT, RF, XGB, GB, and SVC are utilized in this proposed framework to identify COVID patients. Different classification measures such as accuracy, sensitivity, specificity, Kappa index, Matthews correlation coefficient are used to show the efficacy of different classifiers. This study also performed 10-fold cross-validation accuracy to achieve statistical significance using ANOVA, recall rate vs. decision boundary threshold analysis, ROC, and bootstrap ROC. Finally, SHAP analysis is performed to interpret the feature importance and interpret the model. These different classification indicators describe model performance from another point of view. The primary intention to use these indicators is to describe the classification performance from a different perspective. It can be seen from Table 2 that RF yielded the highest classification performance in terms of accuracy, kappa index, and MCC, etc. However, the classification performance of XGB and GBC is very close to RF. The ANOVA and multi-comparison tests show that the average accuracy of RF, XGB, and GBC are very close and are not statistically significant. However, the 10-fold cross-validation accuracy of XGB provides the highest value (see Table 4). Moreover, the balanced XGB model offers the highest classification performance when applied to the original test data (see Table 3). Also, the recall rate vs. decision threshold boundary indicates the superior performance of XGB and SVC (see Figure 9). This concludes that the balanced and optimized XGB model would be the best choice for detecting COVID patients using their inpatient facility data. Therefore, further analyses such as bootstrap ROC and SHAP analysis and features importance analysis are done on a balanced and optimized XGB model.

Regarding the ADASYN algorithm, it should be mentioned that ADASYN adaptively generates synthetic data samples for the COVID-yes class since it is a minority class to reduce bias introduced by imbalanced data distribution. ADASYN moves the classifier’s decision boundary towards harder-to-learn examples, improving the learning performance [28]. Therefore, applying the ADASYN algorithm enhances the learning process and eventually improves our COVID classification performance; see Table 2 to understand the effect of ADASYN in detail. Regarding Bayesian optimization (BO), unlike grid search and random search, it can be mentioned that BO takes the previous objective function evaluation into account, and the function goes to the optimal solution. Therefore, the hyperparameter using BO provides fine-tuning parameters, which ultimately builds an optimized model and consequently increases the classification performance. SHAP is used to determine feature importance and model interpretation; it can be mentioned that SHAP uses a game-theoretic approach, which has an excellent mathematical background and current state-of-the-art approach.

Due to the salient features mentioned above, it can be noted that the proposed framework can not only be applied to COVID-19 detection but also applied to other classification problems such as diabetic prediction, asthma prediction, etc. While describing the significance and strength of this study, it is also logical to explain the weaknesses of this study. The database used in this study is a moderately large dataset. It will be useful to apply the proposed framework on a larger dataset and validate the proposed approach on a completely independent dataset before clinical use. Clinical blood sample data and integration to X-ray and CT-scan will enhance the detection rate and validity. This is beyond the scope of this study.

### A. Development of a Clinically Operable Decision Tree

**FIGURE 15.**
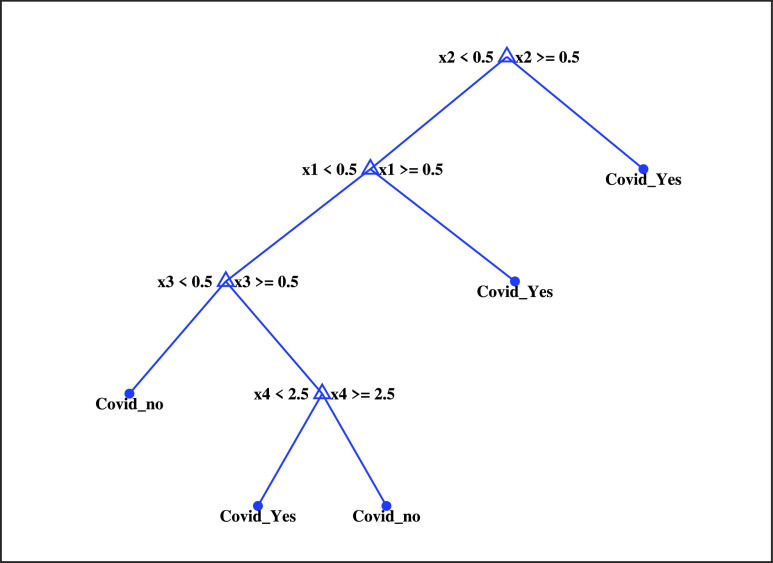
A decision rule using four key features and their thresholds in absolute value.

A clinically operable decision tree would benefit clinical staff as it is straightforward to understand the underlying process. As DT are simple classifiers consisting of sequences of binary decisions organized hierarchically [56], we have built a simple tree by using four important features, $x1\,\,=$ cough; $x2\,\,=$ loss of smell; $x3\,\,=$ high-risk exposure occupation; $x4\,\,=$ sats; Note that, the continuous value of oxygen saturation feature, i.e., $x4$ feature is discretized into three different levels of 1, 2 and 3 to denote severe, moderate and normal level, respectively. $x4$ feature value lies between 75 and 90 mm-Hg is treated as severe, 91 and 95 mm-Hg as moderate, and 96 and 100 mm-Hg as a normal level. Figure 15 represents the corresponding DT, and the description of the tree algorithm is given in Table 6.

**TABLE 6:** Description of the Clinically Operable Decision Tree Algorithm

Node No	Input: x1 = cough; x2 = loss of smell; x3 = high risk exposure occupation; x4 = sats;
Output: The decision of Covid Yes or Covid_no.	
Node 1:	if x2<0.5 then node 2 elseif x2>=0.5 then node 3 else Covid_no
Node 2:	if x1<0.5 then node 4 elseif x1>=0.5 then node 5 else Covid_no
Node 3:	class = Covid_Yes
Node 4:	if x3<0.5 then node 6 elseif x3>=0.5 then node 7 else Covid_no
Node 5:	class = Covid_Yes
Node 6:	class = Covid_no
Node 7:	if x4<2.5 then node 8 elseif x4>=2.5 then node 9 else Covid_no
Node 8:	class = Covid_Yes
Node 9:	class = Covid_no

### B. Development of a Decision Support System (DSS)

**FIGURE 16.**
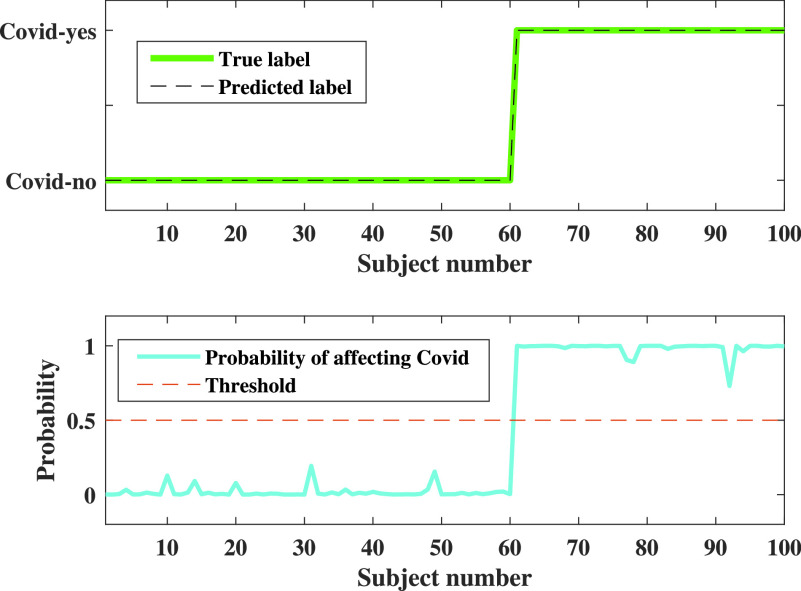
Probabilistic output for the DSS. In the upper figure, the 0 has represented a subject with COVID negative, whereas 1 represented a subject with COVID positive. The lower figure represents a probabilistic outcome of the subject affected by COVID, where the red dotted line defines the threshold level. When the patient data level exceeds this threshold level, then the subject will be considered as COVID positive. Whereas the subject with the probability of less than 0.5, i.e., the threshold value, will be regarded as COVID negative. In either way, we can say that this the chance that a person is affected by COVID.

A DSS could be beneficial to support clinical staff for screening COVID-19 patients from their inpatient facility data. A DSS is usually a graphical representation of decision, COVID-19, in this case, to visualize the probable state of the patient. A possible outcome of COVID suspected patient’s inhouse facility data is presented in Figure 16, in terms of the posterior probability. A probabilistic result is more intuitive to the clinical staff and, therefore, used in this DSS. Note that 100 patients are used from the test database for illustration purposes. The patient is sorted in ascending order so that patients with “COVID-no” labelled appears first, and patients with “COVID-yes” appear.

### C. Comparisons With Other Methods/Studies

To delineate the superiority of our proposed research, an illustrative comparison of our work has been accomplished to other COVID studies. From the tabular illustration [Table 7], it can be mentioned that both Jim *et al.* [11] and Ozturk *et al.* [57] used CNN to obtain the accuracy respectively 92.50% and 98.08%. Furthermore, multiples research works have been carried out by [7], [63], [64] with the direct implementation of XGB using mostly clinical data, where the average of the accuracy obtained from [7], [63] was less than 90%. On top of that, Wu *et al.* [58] used RF to get a classification accuracy of approximately 96%, which outperformed Brinati *et al.* [59], who utilized both DT and RF. In addition, the lowest performance was obtained by Sun *et al.* [60], who used the SVM classifier for clinical and Demographic data. Most importantly, although the accuracy of Wu *et al.* [58] is slightly higher than that of our proposed method, the AUC and Specificity of our work far outweigh the other methodologies mentioned here.

**TABLE 7:** Comparison of Performance With Other Methods

References	Classifiers	Dataset	ACC	SE	SP	AUC
[11]	Deep Convolutional Neural Network	Clinical Image Data	92.5%	94.2%	95.6%	
[58]	RF	Clinical, Demographics	95.95%		96.95%	
[60]	SVM	Clinical, Demographics	77.5%		78.4%	98%
[57]	CNN Darknet	Clinical, Mammographic	98.08%			
[7]	XGB	Clinical, Blood samples of 75 Features	90%			
[61]	Deep learning using LSTM	Demographic	92.67%			
[62]	Logistic Regression and Multinomial NB	Clinical Data	96.2%	96%		
[59]	DT, RF	Hematochemical Values from Blood Exams	86%	95%		
[63]	XGB, RF, DT, SVM	Demographic and Symptom	85%	90%		
[64]	XGB	Clinical Data		92.5%	97.9%	>90%
Proposed	XGB	Inpatient Facility Data	98.50%	99.02%	97.98%	99.4%

## V. Conclusion

This paper presents the optimal use of different machine learning techniques, including state-of-the-art classifiers, to predict COVID. The proposed approach is aimed to handle the real-time in-home dataset in detecting the COVID effectively. Thus, the proposed technique provides a user-friendly and low-cost tool for COVID detection. In designing the method, the COVID dataset, collected from CH-BH, has been used to assess the performance using different classification metrics such as accuracy, sensitivity, specificity, kappa index, etc. The hyper-parameters of different classifiers have been optimized using Bayesian optimization, and the ADASYN has been used to balance the dataset. Compared to the studies presented in this study, it is evidenced that both the classification accuracy and AUC for our proposed framework has attained the highest values of 98.50% and 99.40% using XGB, respectively. As the proposed approach has been applied to a moderately large dataset, it should be used on a big dataset before clinical trials. However, our primary intention is to test the feasibility of such settings. A similar approach can be applied to design other classification problems. Finally, two potential applications of our proposed technique, namely clinically operable decision tree and decision support system, would be beneficial for clinical staff and building an efficient recommender system. It could easily be integrated into mobile devices which would be very useful for the end-users.

## Data Availability

The raw dataset can be accessed through Github: https://github.com/mdcollab/covidclinicaldata. The processed data can be obtained from the first author (Md Abdul Awal; m.awal@ece.ku.ac.bd) of this paper.
